# Deep Learning-Based Evaluation of Maxillary Dental Midline Deviation on Orthodontic Frontal Photographs

**DOI:** 10.3390/bioengineering13060687

**Published:** 2026-06-15

**Authors:** Sercan Taskin, Serra Aksoy, Mine Gecgelen Cesur, Pinar Demircioglu, Ismail Bogrekci

**Affiliations:** 1 Department of Orthodontics, Faculty of Dentistry, Aydin Adnan Menderes University, Aydin 09010, Turkey; sercan.taskin@adu.edu.tr (S.T.); mine.gecgelen@adu.edu.tr (M.G.C.); 2 Department of Computer Science, Ludwig Maximilian University of Munich (LMU), Oettingenstrasse 67, 80538 Munich, Germany; 3 Department of Mechanical Engineering, Faculty of Engineering, Aydin Adnan Menderes University, Aydin 09010, Turkey; pinar.demircioglu@adu.edu.tr (P.D.); ibogrekci@adu.edu.tr (I.B.)

**Keywords:** orthodontics, dental midline, artificial intelligence, deep learning, machine learning, YOLOv8, object detection

## Abstract

**Aim:** This study aimed to detect the maxillary dental midline region on orthodontic frontal photographs using a YOLOv8-based deep learning approach and to evaluate how the detection outputs affect the classification performance of various machine learning algorithms in distinguishing symmetric from asymmetric midline conditions. **Materials and Methods:** A total of 146 standardized frontal photographs (72 with midline deviation ≥ 2 mm from the facial midline, defined by the soft-tissue nasion–subnasal line; 74 symmetric) were analyzed. YOLOv8 was used to obtain bounding-box and keypoint predictions, which were converted into a numerical feature vector and used to train 11 classifiers (including Naive Bayes, Logistic Regression with L1 and ElasticNet penalties, Support Vector Machine, AdaBoost, and others). Performance was assessed using accuracy (with 95% Wilson confidence intervals), precision, recall, F1-score, and ROC-AUC. Optimization of hyperparameters for the downstream classifiers employed five-fold cross-validation along with grid search inside the training data set (n = 126) while final classifier assessment was done using a reserved test data set (n = 20). As the YOLOv8 object detector was trained using the full image dataset before extracting features, the classification metrics presented here should be considered as exploratory results only. **Results:** YOLOv8 achieved mAP@0.5 = 0.995 for midline detection. Naive Bayes attained the highest classification accuracy of 75% (95% CI: 53–89%) with ROC-AUC = 0.75. AdaBoost achieved 65% (95% CI: 43–82%). Several models defaulted to majority-class prediction (accuracy = 40%), indicating insufficient feature discriminability. **Conclusions:** YOLOv8 detected the maxillary dental midline under the present internal experimental conditions. However, because leakage-free outer k-fold validation of the complete detection-plus-classification pipeline was not performed, the classification results should be considered preliminary. Future work should address information leakage, incorporate facial reference frame normalization, include inter-observer reliability assessment, and validate the approach on larger datasets.

## 1. Introduction

Identification of the dental midline is an essential step in the orthodontic diagnostic process, taking into consideration its vital role in terms of aesthetics and functionality. In routine dental practice, such an evaluation is usually performed with the aid of frontal photographs. However, when practitioners try to define the dental midline from two-dimensional images, some parameters, including the positioning of the patient’s head, the expression on his or her face, the overall picture quality, and the level of the practitioner’s skill, could affect the procedure. Also, assessments relying on manual landmarks placement are vulnerable to intra-observer and inter-observer variability [1,2].

However, research has found that there is usually a concurrence of facial and maxillary midline interincisal in most people, with a success rate of about 94.3% [3]. While it is acceptable to have slight misalignments, larger deviations surpassing the 2 mm margin are not acceptable from an aesthetic standpoint for either professional or laypeople [4]. This effect is particularly noticeable when dealing with patients having asymmetric faces, which makes things even more complicated [5,6].

Conventional photographic analysis methods require the manual identification of dental and facial reference points, making the evaluation process time-consuming and difficult to standardize. Observer-dependent variability in symmetry and midline assessments on frontal photographs has been reported at clinically significant levels [7,8]. These limitations have increased the need for more objective, reproducible, and automated methods in orthodontic image analysis.

The dependence on manually placed landmarks like subnasale/philtrum takes a lot of time, while at the same time being highly susceptible to errors made by humans. Inter-observer variability was found to occur significantly, where some cases have reported that midlines have shifted in up to 34.5% of orthodontic patients, due mainly to the variation in patients’ head positions or photographic quality [5,6].

Recent advances in deep learning-based image processing have enabled the automatic detection of anatomical structures in medical and dental images. Deep learning models learn hierarchical feature representations directly from raw image data without requiring predefined rules or manual feature extraction, offering an advantage over conventional approaches for datasets with high variability, such as orthodontic photographs [9]. Convolutional Neural Networks (CNNs) prove themselves to be particularly adept at recognizing the presence of localized objects within images through the process of gradually becoming more complex with each stage of the learning process, beginning with edge detection and eventually moving on to texture recognition and recognition of the geometry that makes up different forms. One application area where CNN-based systems have made particular strides is orthodontics [10,11].

Among single-stage object detection architectures, the You Only Look Once (YOLO) family is notable for its end-to-end trainable structure and high computational efficiency [12]. YOLOv8, the architecture employed in the present study, features an anchor-free detection paradigm, an improved feature pyramid network, and native support for keypoint (pose) estimation, providing performance advantages over earlier versions [13]. The reasoning behind choosing the YOLOv8 among other models lies in the fact that the model itself possesses the capacity to perform key point detection, performs extremely well in object detection tasks, and provides an open-source implementation (Ultralytics), thus offering an end-to-end solution for detection and features extraction. This aspect can be useful in automatically detecting small objects with distinct anatomy like maxillary dental midlines. Recent comprehensive reviews have documented the expanding applications of YOLO-based models in medical and dental image analysis [14,15,16]. Furthermore, YOLO-based models directly produce bounding-box and coordinate outputs that can serve as numerical features in subsequent analytical stages [17].

Moreover, the quantitative efficacy of the YOLO model in current orthodontic investigations emphasizes its suitability for midline recognition in an automated fashion. Specifically, the latest version of the YOLO family—namely, YOLOv11—has been reported to exhibit an mAP score of 91.8% when detecting shifts in dental midlines in standardized photos [18]. Alongside its remarkable precision, the YOLO architecture is also highly efficient in terms of landmark localization, with YOLOv8-powered solutions having shown their ability to detect landmarks within the range of 2 mm to 3 mm from their true position reliably [19,20]. Such levels of accuracy are particularly important due to the fact that discrepancies from midline position larger than 2 mm indicate an aesthetically unpleasing asymmetry perceived even by people without professional training in the field [3,4]. Through their provision of highly accurate bounding box and keypoint locations, YOLO-based frameworks provide a strong basis for the development of hybrid systems for standardized classification of dental asymmetries [18,21].

Most studies in the literature have focused on determining how well the models based on deep learning perform object and event detection and recognition tasks. On the other hand, little attention has been paid to examining the extent to which detection outcomes help subsequent classification processes [22]. Studies examining the impact of deep learning-based dental midline detection outputs, used in conjunction with classical machine learning algorithms, on symmetry–asymmetry classification remain scarce [23]. Hybrid approaches that combine deep learning with classical machine learning methods hold potential for providing automated, reproducible, and computationally efficient solutions in orthodontic image analysis [24].

The technical verification of such hybrid models often depends on the efficiency of the employed detection technique itself. The recent evaluation of the performance of YOLOv8 against its predecessors, like YOLOv5 in dental segmentation tasks, revealed that YOLOv8 yielded better results by attaining more precision (0.913) and F1-scores (0.931) [25]. Furthermore, single-stage detection mechanisms appear to be relatively more efficient than their two-stage counterparts in terms of accuracy. As evidence of this, single-stage mechanisms have been shown to attain a mean average precision (mAP) as high as 94.30% in complex dental classification tasks [26]. However, apart from high accuracy, there is another important factor which increases the clinical applicability of such systems. Automated deep learning techniques have been shown to significantly improve the speed and ease of conducting orthodontic diagnosis by reducing the need to spend 30 min analyzing patient records to merely 30 s [27,28].

This paper intended to detect and localize the midline region of the maxillary teeth from orthodontic images using an automatic model with YOLOv8 architecture. In addition to that, this work sought to explore how the outputs of this detection algorithm affected the performance of various machine learning models when they classified midline regions into either symmetric or asymmetric.

## 2. Materials and Methods

### 2.1. Study Design

This study was designed as a methodological, cross-sectional, image-based artificial intelligence investigation aimed at automatically detecting the maxillary dental midline region on standardized orthodontic frontal photographs using a deep learning-based object detection framework and subsequently evaluating the effect of these detection outputs on the classification performance of various machine learning algorithms.

No clinical intervention was performed and no direct contact with patients occurred during the study. All analyses were conducted retrospectively on digital photographic records from the institutional orthodontic archive. As the study focused on computational analysis of anatomical features from images, no patient-centered clinical outcomes were measured.

The methodological workflow consisted of four main stages: (1) dataset assembly and case selection, (2) image preprocessing and manual annotation of the maxillary dental midline region, (3) automatic detection of the annotated region using the YOLOv8 deep learning architecture, and (4) classification of symmetric and asymmetric maxillary dental midline conditions using numerical features derived from the detection outputs of the trained model.

The study should be interpreted as an AI-assisted diagnostic support investigation rather than as an interventional clinical study.

### 2.2. Ethical Approval

The study protocol was approved by the Aydın Adnan Menderes University Faculty of Medicine Non-Interventional Clinical Research Ethics Committee on 29 April 2024 under decision number 527894. The study was conducted in accordance with the principles of the Declaration of Helsinki. Informed consent was waived by the ethics committee due to the retrospective, non-interventional nature of the study and the exclusive use of de-identified clinical photographs.

### 2.3. Sample and Dataset

The dataset consisted of standardized frontal orthodontic photographs obtained from individuals whose pre-treatment records were available in the archive of the Department of Orthodontics, Faculty of Dentistry, Aydın Adnan Menderes University. A total of 146 frontal photographs from 146 individuals were included in the final dataset. Each image corresponded to a single individual, and no duplicate images from the same participant were used to avoid repeated sampling bias.

Among the included cases, 72 individuals exhibited maxillary dental midline deviation, whereas 74 individuals were classified as having a symmetric maxillary dental midline. This approximately balanced distribution was maintained to reduce class-related bias during model training and evaluation. The dataset was partitioned as follows: a training set (n = 126) was used for model development, and a fixed held-out test set (n = 20; 8 symmetric, 12 asymmetric) was reserved for final performance evaluation (Table 1). This fixed partition method was employed in the current study when testing the performance of the exploratory classification model. However, one must note that this cannot be considered to be a proper k-fold cross-validation of the entire YOLOv8 detection/classification process since the detector is trained only once.

This fixed 126/20 partition was used for exploratory classifier evaluation. It should not be interpreted as a full k-fold validation of the complete YOLOv8 detection-plus-classification pipeline, because the detector was not re-trained separately within each outer fold. The study population comprised individuals in the permanent dentition period, as this dentition stage allows more reliable identification of the maxillary interincisal midline and reduces uncertainty related to transitional dentition. Only pre-treatment records were included so that orthodontic appliances, treatment-induced tooth movement, or soft tissue changes secondary to treatment would not influence image interpretation.

#### 2.3.1. Inclusion Criteria

The inclusion criteria were as follows: availability of a pre-treatment frontal orthodontic photograph in the institutional archive, presence of permanent dentition, photograph quality sufficient for visual identification of the maxillary central incisors and the maxillary dental midline region, frontal image orientation compatible with orthodontic photographic evaluation, and absence of substantial image distortion or obstruction in the region of interest.

#### 2.3.2. Exclusion Criteria

The research ruled out individuals from the study based on a number of considerations regarding the photographs used. The images had no blurring, were of high quality, and showed adequate lighting. Individuals whose photographs showed some form of head movement or tilting of the head were not considered for the study. Additionally, images with a clear view of the maxillary central incisors were included in the research.

The photographs used in this study were all captured using standard orthodontic photography guidelines. The subject position in the photographs was with the head in its natural posture, closed lips, and no smile. Only those photographs where the maxillary dental midline region was interpretable were included in the study.

The dataset consists of 146 images, forming a set of standardized front views that have been selected following strict quality standards. Despite the relatively low sample size, it is comparable with what is usually observed in preliminary studies in the field of artificial intelligence applications in dentistry. To compensate for some of the issues associated with using a small sample size, techniques such as data augmentation were used in YOLOv8 training, and five-fold cross-validation was performed when optimizing the classifier. The selected methods partly mitigate the issues associated with sample size. Nonetheless, the low number of images inevitably limits the generalizability and statistical power of the results.

The classification target (symmetric vs. asymmetric maxillary dental midline) was defined with reference to the facial midline, operationalized as the vertical line connecting the soft-tissue nasion to the subnasal point on the frontal photograph. Midline deviation was quantified as the horizontal distance between the maxillary interincisal contact point and this facial midline. Cases with a deviation ≥ 2 mm were classified as asymmetric; cases with a deviation < 2 mm were classified as symmetric. This threshold is consistent with values reported in the orthodontic literature as the perceptibility limit for dental midline discrepancies [8]. All ground-truth labels were assigned by a single experienced orthodontist (S.T.) on the basis of these predefined anatomical criteria.

#### 2.3.3. Photographic Standardization and Available Metadata

The photographs used for the analysis were acquired through retrospective selection from the institutional orthodontic photographic archive and conform to criteria set for taking photographs of the patient’s face from a frontal view. Criteria considered were natural position of the patient’s head, mouth with closed lips, lack of smiles, adequate lighting, and the presence of clearly visible maxillary central incisors. Images which contained any form of blurriness, head tilt, poor lighting, obfuscation of the region with the maxillary incisors, and geometric distortion were excluded. The fact that these were retrospective images meant that information on their capture such as camera models, type of lens, subject distance to the camera, lighting arrangement, file type, and camera settings were not always available.

### 2.4. Image Preprocessing and Annotation

**Figure 1:**
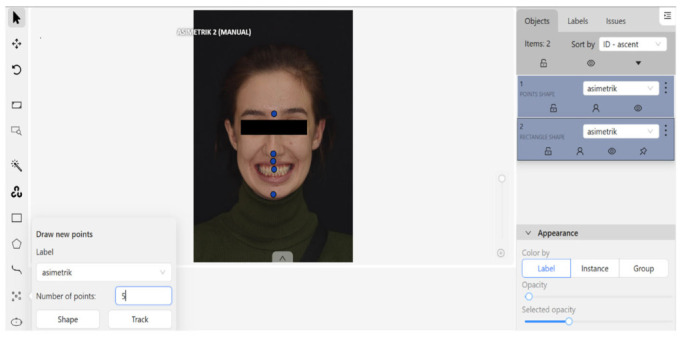
Labeling of the Five Keypoints used for Midline Detection with CVAT. Blue dots indicate the five annotated keypoints (1–5) used for midline detection.

Every stage of preprocessing and annotation process was done within the Python 3.14 program. Prior to performing any annotation and training of the models, all images had to be put into the same unified digital form to ensure that everything runs smoothly in the selected deep learning framework. This was done by standardizing the resolution, structuring the files correctly, and normalizing the images. All this was done using OpenCV 4.11.0 and NumPy 2.2.2 libraries, thereby reducing variations arising from different sizes, scales, and lighting of the images (Figure 1).

**Figure 2:**
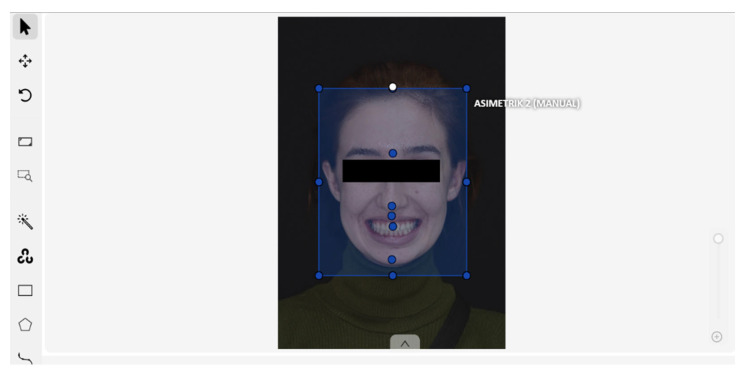
Annotation of bounding boxes used for maxillary dental midline detection performed using CVAT. The marker/box indicates the annotated maxillary dental midline bounding-box region.

The maxillary dental midline region in each image was manually annotated using bounding boxes. Annotation was performed in CVAT (Computer Vision Annotation Tool). The region of interest was defined on the basis of the interincisal area between the maxillary central incisors, representing the clinically relevant location of the maxillary dental midline. All labels were created in a format compatible with the YOLO training pipeline (Figure 2).

In addition to bounding boxes, five anatomical keypoints were annotated for each image. The keypoints were defined as follows: (1) the mesial incisal edge of the right upper central incisor, (2) the distal incisal edge of the right upper central incisor, (3) the interincisal contact point between the upper central incisors, (4) the mesial incisal edge of the left upper central incisor, and (5) the distal incisal edge of the left upper central incisor.

To reduce annotation inconsistency, all labelling procedures were performed by a single operator (S.T.) according to predefined anatomical criteria. The same annotation logic was applied across all images, and each annotation was visually reviewed before inclusion in the training dataset.

This methodology helped maintain internal consistency; however, subjectivity is a potential pitfall. To address that, we are designing a subsequent experiment where the intra-rater reliability would be evaluated by repeating annotation of a randomly selected 20% of the sample four weeks later. In addition, inter-observer agreement would be studied, and the agreement regarding the classes will be measured by Cohen’s kappa coefficient, while ICC would be used to estimate the agreement on keypoint annotations. This is a critical step for validation of the ground truth.

### 2.5. Deep Learning Based Detection: YOLOv8

Automatic detection of the maxillary dental midline region was performed using the YOLOv8 pose-estimation architecture, a single-stage deep learning based object detection model with native support for simultaneous bounding-box prediction and keypoint estimation [13]. The model was implemented using the Ultralytics YOLOv8 pose-estimation framework with a PyTorch 3.11 backend in the Python environment. The YOLOv8s-pose model variant (yolov8s-pose.pt) was used for bounding-box and keypoint detection.

The following data augmentation techniques were applied during training: HSV augmentation (hue ± 0.015, saturation ± 0.7, value ± 0.4), scale augmentation (±0.5), translation (±0.1), erasing augmentation (probability = 0.4), and mosaic augmentation (probability = 1.0). Horizontal flipping was disabled (fliplr = 0.0) because the dataset contained paired left–right keypoints, and horizontal flipping would invert the anatomical direction of asymmetry. These augmentations were applied to increase the effective diversity of the training set and reduce the risk of overfitting given the limited dataset size. The model was configured for 1000 epochs with a batch size of 32, an input image size of 640 × 640 pixels, and the AdamW optimiser with momentum = 0.9 and weight decay = 0.0005. Early stopping with a patience of 100 epochs was applied; training was automatically terminated at epoch 370, with the best results observed at epoch 270.

For the YOLOv8 detection stage, all 146 images were provided in a single dataset directory, and the Ultralytics framework generated an internal training/validation split during detector training. For the downstream classification task, the images were partitioned into a training set (n = 126) and a fixed held-out test set (n = 20; 8 symmetric, 12 asymmetric). It is important to note that YOLOv8 was trained using the full 146-image dataset before feature extraction. Therefore, the 20-image classifier test set was not independent at the detector level, because the detector had already seen these images during its own training workflow. The five-fold cross-validation used during classifier optimization does not correct this detector-level information leakage, because it was applied only to the downstream classifier stage and not to the complete YOLOv8 detection-plus-classification pipeline. Consequently, the classification results reported in this study should be interpreted as exploratory proof-of-concept findings rather than as unbiased estimates of full-pipeline generalization.

Detection performance was evaluated using F1-score, mean average precision (mAP), and recall. An Intersection over Union (IoU) threshold of ≥0.5 was applied, following standard practice in the object detection literature [29].

### 2.6. Feature Extraction and Data Representation

Bounding-box and keypoint outputs from the trained YOLOv8 model were converted into a numerical feature vector for each image. The feature vector comprised 15 dimensions: normalized bounding-box parameters (centre x, centre y, width, height; 4 values), the detection confidence score (1 value), and normalized coordinates of the five keypoints (kp1_x, kp1_y through kp5_x, kp5_y; 10 values). All coordinates were in the [0, 1] range relative to image dimensions, as per YOLO convention.

All coordinates were in the [0, 1] range relative to image dimensions, as per YOLO convention. No additional normalization to a facial anatomical reference frame was applied; this design choice and its implications are discussed in Section 4.

The feature extraction process involved the use of Python programming language with extensive reliance on NumPy and Pandas library modules. The resulting data set was arranged in the form of a feature matrix wherein each row represented an image and each column consisted of a numerical feature extracted from the detection results. This feature matrix was utilized as input in the classification phase of the machine learning model.

### 2.7. Machine Learning Models

In the process of classification, several machine learning techniques under supervision have been used for the classification of two cases of the dental midline in the maxilla, which may be either symmetric or asymmetric. This decision was made according to the numerical data obtained from the outputs of YOLOv8.

The evaluated algorithms were: Naive Bayes (GaussianNB), Logistic Regression, Logistic Regression with L1 penalty, Logistic Regression with ElasticNet penalty, K-Nearest Neighbours, Decision Tree, Random Forest, Support Vector Machine (RBF kernel), AdaBoost, Extra Trees, and Linear Discriminant Analysis.

The use of different types of classification models allowed us to perform an extensive comparison of the different learning methods involved. These include probability models, linear models, distance models, tree models, ensemble models, and margin models. The importance of using a variety of classification algorithms was due to the fact that we could not determine beforehand which type of algorithm would work best for classifying image anatomical characteristics.

To provide a reference for evaluating whether the machine learning models add value over a simple geometric rule, a baseline classifier was implemented. This classifier computed the normalized horizontal offset of keypoint 3 (interincisal contact point) from the image center and applied a fixed threshold: cases with an offset exceeding the threshold were classified as asymmetric; all others were classified as symmetric. The threshold was optimized on the training set.

### 2.8. Validation Strategy

For the downstream classification task, the dataset was divided into a training set of 126 images and a fixed held-out test set of 20 images, including 8 symmetric and 12 asymmetric cases, using stratified random sampling. Five-fold cross-validation combined with grid search was performed only within the training set to optimize the hyperparameters of the machine learning classifiers. After the optimal hyperparameters had been selected, each classifier was re-trained using all 126 training images and then evaluated on the fixed 20-image test set.

This validation strategy should be interpreted as an internal classifier-level evaluation rather than as an outer k-fold validation of the complete YOLOv8 detection-plus-classification pipeline. Specifically, YOLOv8 was not re-trained separately for each outer fold, meaning that the fixed test images were not fully independent at the detector level. Therefore, the reported confusion matrices are based on the 20-image classifier test set and should not be interpreted as aggregated k-fold confusion matrices across all 146 images. A fully leakage-free evaluation of the complete pipeline would require re-training YOLOv8 within each outer fold, so that each held-out fold remains unseen by both the detector and the classifier.

### 2.9. Performance and Statistical Analysis

The predictive performance of the machine learning models was evaluated using accuracy, precision, recall, F1-score, and the area under the receiver operating characteristic curve (ROC-AUC). For each metric, 95% Wilson confidence intervals were computed to reflect the limited test-set size (n = 20). Accuracy was defined as the proportion of correctly classified instances among all samples. Because accuracy may be insensitive to class-specific error patterns, it was interpreted together with the remaining performance metrics. Precision was calculated as the proportion of true positive predictions among all predicted positive cases. Recall (sensitivity) was defined as the proportion of actual positive cases correctly identified by the model; this metric was considered particularly important for assessing whether asymmetric cases were missed. The F1-score was calculated as the harmonic mean of precision and recall, providing a balanced summary of both false-positive and false-negative tendencies.

The ROC-AUC metric was used to quantify the overall discriminative ability of each classifier across multiple threshold values. ROC curves were generated from the available classifier score outputs. Because the AdaBoost ROC curve showed inconsistency with the label-based classification metrics, the AdaBoost ROC-AUC was interpreted with caution and was not used as the basis for comparative conclusions. An ROC-AUC score close to 1.0 indicates very high discrimination capabilities, while values closer to 0.5 indicate results comparable to those obtained by random guessing. To analyze and compare performance metrics of models, ROC curves, confusion matrix plots, and various types of comparison plots were created using scikit-learn along with Matplotlib 3.10.0. In all experiments, the same feature set and methodology was employed for each model tested.

## 3. Results

The results have been presented in two separate phases, where initially the detection capabilities of the YOLOv8 algorithm have been analyzed, followed by evaluating the classification capabilities of different machine learning algorithms using characteristics generated through the output of the detection phase.

### 3.1. Detection Performance of the YOLOv8 Algorithm

The maxillary dental midline detection task was found to be very successful using YOLOv8. In order to measure the accuracy of the detector’s detections, threshold-dependent and threshold-independent measures were used, which included F1 score, precision, recall, and mean average precision. From the analysis of the F1-confidence graph, the F1-score remains close to 1.0 until a confidence threshold of ~0.88, after which it decreases due to the decrease in true positives. The precision increases with increasing confidence, reaching 1.0 at a confidence level of ~0.936 and maintaining this value, showing that there are no false positives.

**Figure 3:**
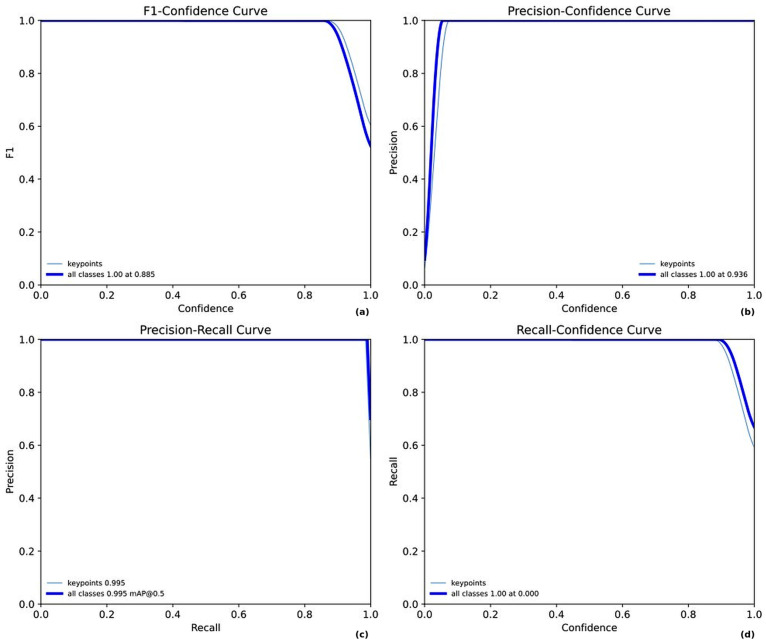
Performance curves of the YOLOv8 model: (**a**) F1-score curve, (**b**) Precision–Confidence curve, (**c**) Precision–Recall curve, and (**d**) Recall–Confidence curve.

The mean average precision calculated at an Intersection over Union threshold of ≥0.5 was mAP@0.5 = 0.995, indicating a high level of overlap between predicted bounding boxes and manually annotated ground-truth regions. This high localization performance should be interpreted in context: the maxillary interincisal region is a high-contrast, well-defined anatomical landmark on standardized photographs, and near-perfect localization under these conditions is expected rather than exceptional. Both training and validation loss curves showed a consistent decline across epochs, with no widening divergence, indicating stable model convergence without overfitting (Figure 3).

### 3.2. Classification Performance of Machine Learning Models

Features derived from the YOLOv8 bounding-box and keypoint outputs (15-dimensional feature vector) were used to train classifiers for distinguishing symmetric from asymmetric dental midline conditions. Hyperparameter optimization was performed using five-fold cross-validation with grid search on the training set (n = 126).

Classifier performance was evaluated on the fixed held-out test set (n = 20; 8 symmetric, 12 asymmetric). However, because the YOLOv8 detector had been trained using the full image dataset before feature extraction, these results should be interpreted as exploratory classifier performance based on YOLO-derived features, not as unbiased full-pipeline generalization performance. Results are summarized in Table 2.

As far as the models under analysis are concerned, Naïve Bayes performed exceptionally well as it scored the highest accuracy of 0.75 along with a 95% Wilson confidence interval between 0.53 and 0.89. The model also demonstrated impressive results in class-related metrics, scoring a precision value of 0.64, a recall rate of 0.88, and an F1 score of 0.74 for symmetric class. In case of asymmetric class, the model delivered a precision of 0.89, recall of 0.67, and an F1 score of 0.76. The ROC-AUC was 0.75. AdaBoost achieved an accuracy of 0.65 (95% CI: 0.43–0.82).

For the symmetric class, precision, recall, and F1-score were 0.54, 0.88, and 0.67, respectively; for the asymmetric class, these values were 0.86, 0.50, and 0.63. The macro and weighted F1-scores were both 0.65. Extra Trees achieved an accuracy of 0.60 (95% CI: 0.39–0.78). When the results of the metrics are considered according to classes individually, the precision, recall, and F1-score in the case of the symmetric class amount to 0.50 each. In the asymmetric case, all three measures have the same value of 0.67. If the results are calculated in accordance with aggregated classes, the macro F1-score amounts to 0.58, whereas the weighted F1-score equals 0.60.

Logistic Regression achieved an accuracy of 0.55 (95% CI: 0.34–0.74), with precision, recall, and F1-score of 0.46, 0.75 and 0.57 for the symmetric class, 0.71, 0.42 and 0.53 for the asymmetric class. K-Nearest Neighbours achieved an accuracy of 0.50 (95% CI: 0.30–0.70), with precision, recall, and F1-score of 0.40, 0.50, and 0.44 for the symmetric class, and 0.60, 0.50 and 0.55 for the asymmetric class. Random Forest, Decision Tree and Linear Discriminant Analysis each achieved an accuracy of 0.45 (95% CI: 0.26–0.66), with ROC-AUC values between 0.41 and 0.45, indicating limited discriminative ability.

**Figure 4:**
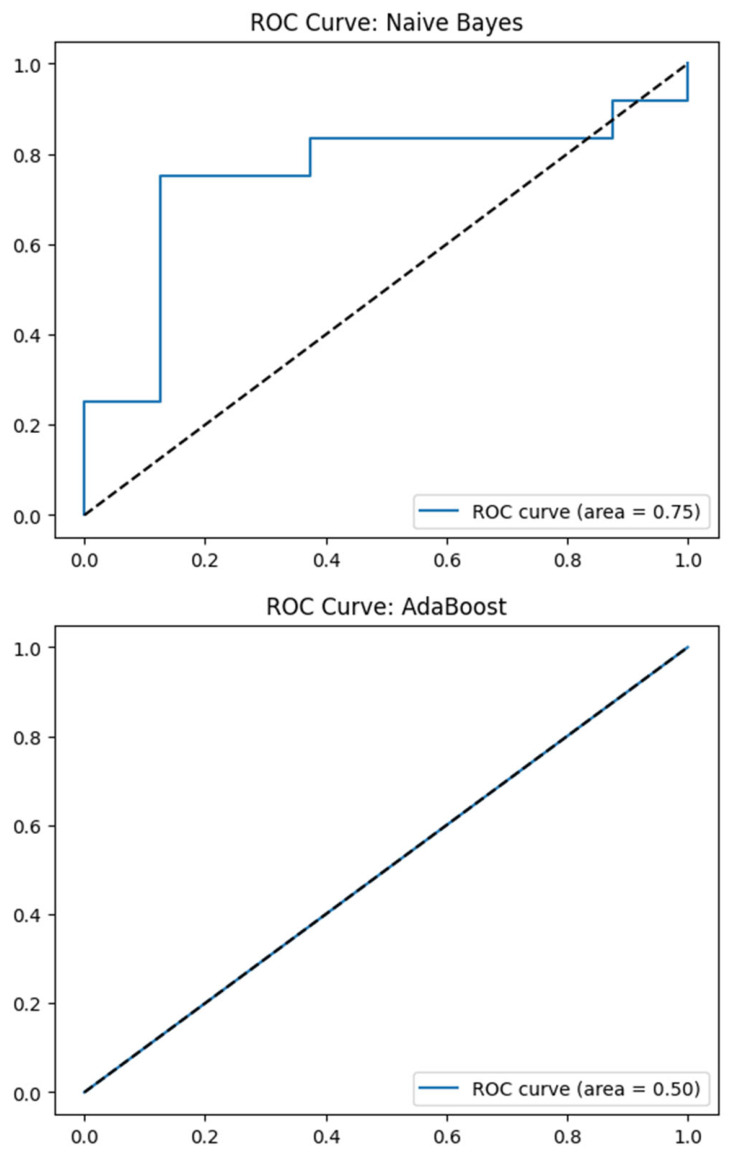
Receiver operating characteristic (ROC) curves for the Naive Bayes and AdaBoost models. The AdaBoost ROC-AUC should be interpreted with caution because of inconsistency between the ROC curve and label-based classification metrics.

Support Vector Machine (RBF kernel), Logistic Regression with L1 penalty, and Logistic Regression with ElasticNet penalty all achieved identical metrics: an accuracy of 0.40 (95% CI: 0.22–0.61), precision of 0.40 and recall of 1.00 for the symmetric class, and F1-score of 0.57, with all-zero metrics for the asymmetric class. This identity across three structurally different algorithms indicates that all three defaulted to predicting the majority class (symmetric) for all test instances, providing direct evidence that the current feature representation has insufficient separability for the classification task. ROC and confusion matrix figures are shown only for Naive Bayes and AdaBoost as illustrative examples. Extra Trees is reported numerically in Table 2 but was not included in the figures. ROC and confusion matrix figures are shown only for Naive Bayes and AdaBoost as illustrative examples. Extra Trees is reported numerically in Table 2 but was not included in the figures. ROC curve analysis of the Naive Bayes and AdaBoost models is given in Figure 4.

### 3.3. ROC Curve and Confusion Matrix Analysis

ROC curves were used to determine the effectiveness of the classification model when different threshold levels were applied. The ROC curve was plotted based on the true positive rate plotted against the false positive rate using the different cutoffs. ROC curves were generated using the available classifier score outputs. The AdaBoost ROC curve showed inconsistency with the label-based metrics and is therefore interpreted cautiously. The Naive Bayes model had a value of ROC-AUC equal to 0.75, which is evident of a strong separation that occurred between the true positive rate and false positive rate throughout the entire decision threshold range. On the other hand, logistic regression had a ROC-AUC of 0.64, demonstrating moderate discriminate power. The KNN approach resulted in a ROC-AUC of 0.56, and as such, this model exhibited minimal discriminatory power. For the random forest, decision tree, and linear discriminant analysis approaches, the ROC-AUC values were between 0.41 and 0.45.

The ROC curve produced by the application of the AdaBoost algorithm to the developed model was seen to lie in a close to diagonal trajectory. This gave rise to an AUC value of 0.50. This was unlike the other models used for classification whose accuracy score was seen to be 0.65. The fundamental problem causing this conflict was the improper extraction of probabilities through decision_function rather than predict_proba. The classification metrics in Table 2 are based on predicted labels; however, given the small test set and the leakage limitation described above, these metrics should be interpreted as exploratory rather than confirmatory.

**Figure 5:**
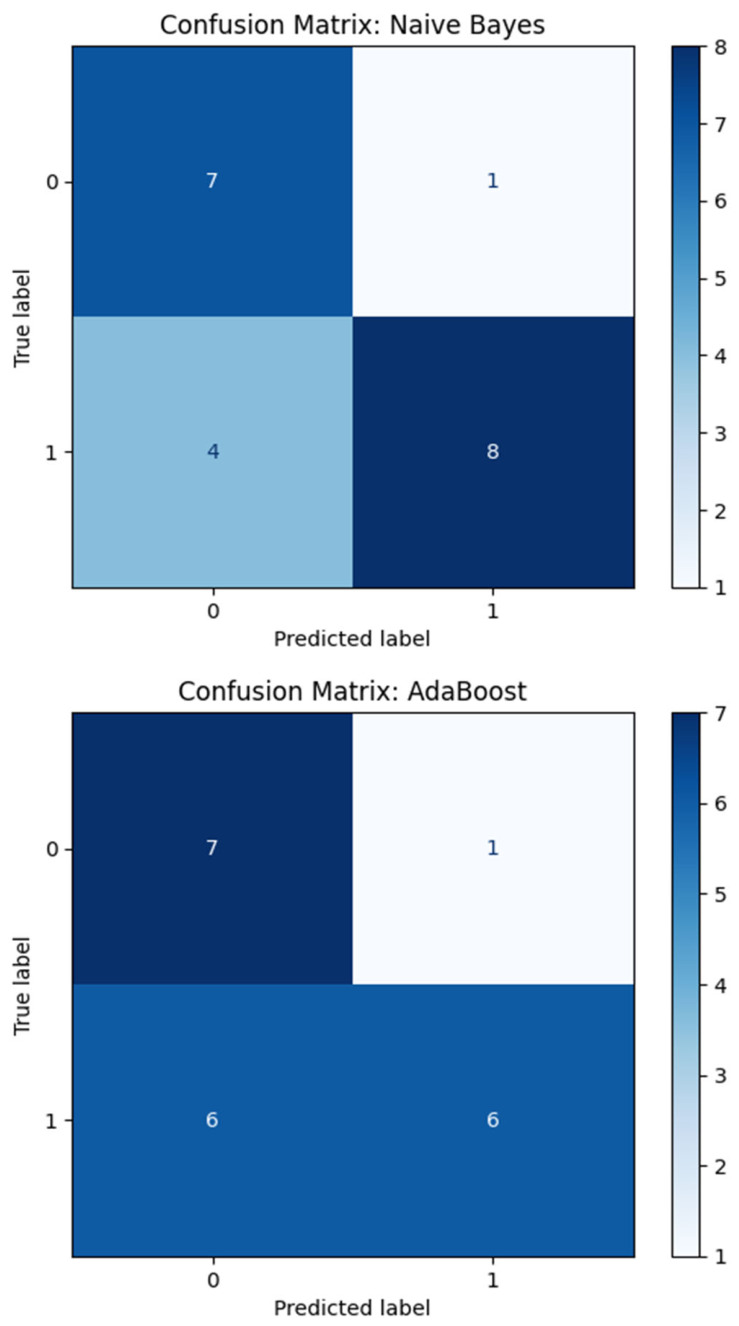
Confusion matrices for the Naive Bayes and AdaBoost models on the fixed 20-image test set, not aggregated across all 146 images. Class labels 0 and 1 indicate symmetric and asymmetric cases, respectively.

Confusion matrices were generated for each model (Figure 5). Naive Bayes correctly classified the majority of both symmetric and asymmetric cases, with misclassifications distributed between false positives and false negatives. AdaBoost showed a similar pattern, with correct classifications in both classes. For Support Vector Machine (RBF kernel), Logistic Regression with L1 penalty, and Logistic Regression with ElasticNet penalty, the confusion matrices showed concentration of all predictions in the symmetric class, consistent with the majority-class prediction behaviour noted above.

### 3.4. Overall Evaluation

The findings indicate that YOLOv8-derived features can be used in downstream classifiers; however, the current results do not support a statistically robust ranking of algorithms. Naive Bayes achieved the highest point estimate of accuracy, but the 95% confidence intervals of the evaluated models overlapped substantially. Therefore, differences between classifiers should be interpreted cautiously.

## 4. Discussion

### 4.1. Principal Findings

This study evaluated the automatic detection of the maxillary dental midline region on orthodontic frontal photographs and the impact of the resulting detection outputs on the classification performance of various machine learning algorithms. YOLOv8 achieved high detection accuracy (mAP@0.5 = 0.995), while the classification of symmetric versus asymmetric midline conditions reached a best accuracy of 75% (Naive Bayes), with a wide confidence interval (53–89%) reflecting the limited test-set size. This high detection accuracy (0.995 mAP) aligns with and even exceeds recent benchmarks in dental object detection literature, which typically report mAP values between 91.8% and 94.30% for multi-class dental tasks [18,26].

### 4.2. Detection Performance

The mAP@0.5 value of 0.995 indicates a high level of agreement between the predicted and manually annotated regions under the present experimental conditions. This finding is consistent with previous studies showing that CNN-based, YOLO-based, and other single-stage object detection models can localize clearly defined anatomical landmarks in dental and facial images [9,30]. Specifically, YOLOv8 has been shown to outperform earlier versions like YOLOv5 in dental segmentation, achieving higher precision scores (0.913) and providing the reliability needed for identifying landmarks within a clinically acceptable 2 mm radius [19,20,25]. However, this result should be interpreted with caution. The maxillary interincisal region is a relatively high-contrast and well-defined anatomical area on standardized frontal photographs; therefore, the high localization performance may partly reflect the controlled nature of the images and the relative simplicity of the detection task.

It should also be noted that the detection performance was calculated using an internal validation split derived from the same limited dataset. Therefore, the reported mAP@0.5 should be regarded as an internal performance estimate rather than as evidence of external or leakage-free detector generalization. This distinction is important because the YOLOv8 outputs were later used as input features for the downstream classification models.

The single-stage architecture of YOLOv8 enables efficient localization of small anatomical structures and generation of bounding-box and keypoint outputs that can be used in subsequent analytical stages [12,17]. The confidence-threshold curves showed that higher confidence thresholds were associated with higher precision and lower recall. This pattern should be considered in future studies when selecting an operational threshold for clinical or research use.

### 4.3. Classification Performance and Feature Limitations

An important finding of this study is that high detection accuracy did not translate into equivalently high classification performance. This disconnect has been reported in prior methodological studies examining the downstream impact of deep learning outputs [22,23]. The two-stage hybrid architecture (detection followed by classification) allows independent evaluation of each stage, revealing that the feature representation, rather than the choice of classifier, is the primary bottleneck. This performance disconnect suggests that raw coordinates lack the discriminative power found in more integrated diagnostic systems [21].

Naive Bayes achieved the highest point estimate among the evaluated classifiers; however, the wide and overlapping confidence intervals prevent a statistically conclusive ranking of model performance. This is consistent with classical machine learning theory, which holds that simpler models generalize better on small datasets with limited feature discriminability [24,31]. The relatively small sample size (146 images, 20 test cases) and the low dimensionality of the feature vector (15 features) likely favoured models that assume conditional independence over more flexible but data-intensive approaches.

AdaBoost and Extra Trees achieved moderate point estimates. However, because of the small test set and overlapping confidence intervals, these apparent differences should not be interpreted as evidence of statistically meaningful superiority or inferiority between models. The moderate performance of Logistic Regression and K-Nearest Neighbours similarly suggests that both linear and distance-based approaches are unable to fully capture the variability between symmetric and asymmetric cases in this feature space.

Support Vector Machine (RBF kernel), Logistic Regression with L1 penalty, and Logistic Regression with ElasticNet penalty all defaulted to majority-class prediction, achieving identical metric profiles with perfect recall for the symmetric class and zero detection of the asymmetric class. This behaviour across three structurally different algorithms provides direct evidence that the current feature representation has insufficient separability. Similar findings have been reported in studies on classification of low-variance medical datasets [32]. The higher recall for the symmetric class relative to the asymmetric class observed across most classifiers suggests that the extracted features capture symmetry more effectively than the specific patterns of asymmetry.

### 4.4. Limitations

#### 4.4.1. Absence of Facial Reference Frame Normalization

The most important methodological limitation of this study is that the features used for classification were not normalized to a facial reference frame. The YOLO-normalized bounding-box and keypoint coordinates are relative to image dimensions, meaning that variations in framing, camera-to-patient distance, head tilt, and cropping can change coordinate values without reflecting any change in the underlying clinical asymmetry. This is a probable explanation for the modest classification performance and substantially limits the validity of the current approach. Future work should normalize features to a facial reference frame, for example by expressing the midline offset as a proportion of interpupillary distance or facial width. Addressing this is vital because the clinical perception of a midline shift is deeply influenced by the surrounding facial symmetry, with 2 mm being the established threshold at which aesthetic disharmony becomes noticeable to both professionals and patients [3,5,6].

#### 4.4.2. Information Leakage Between Detection and Classification

The trained YOLOv8 model has been tested with all 146 images, and then the output of the bounding boxes and keypoints generated by this model has been used as the input features for classifying the same set of images. Thus, the trained YOLOv8 model is already aware of the images used as test data for classifying them. This will result in information leakage from detection to classification. In fact, even though the classification model has been optimized using five-fold cross-validation, it has been done only at the downstream step and not at the end-to-end level comprising both the detection and classification steps. As a result, it will produce an optimistic estimate of the classifier’s performance. A better design will involve either training the YOLOv8 model on only the train part and then testing on the untouched test part or using an outer cross-validation framework where each fold is kept unseen by both models. Systematic reviews have highlighted that such methodological rigor is essential to avoid the high risk of bias often found in preliminary deep learning studies in dentistry [33,34].

#### 4.4.3. Single-Observer Annotation

All ground-truth annotations were performed by a single operator. While this ensures internal consistency, it does not allow assessment of inter-observer agreement, and intra-observer repeatability was not formally tested. Given that the entire downstream analysis depends on the validity and consistency of these labels, this is a substantive limitation. Future work should include intra-observer repeatability testing and inter-observer agreement with at least one additional orthodontist, reported using Cohen’s κ for class labels and intraclass correlation coefficients for keypoint coordinates.

#### 4.4.4. Small Test Set and Statistical Power

The test set comprised only 20 images (8 symmetric, 12 asymmetric). The 95% Wilson confidence interval for the best accuracy (75%) spans 53–89%, encompassing most values reported across all models. With 11 classifiers benchmarked on 20 images, the experiment has limited statistical power to detect meaningful performance differences. Conclusions regarding the relative merits of individual algorithms should therefore be considered tentative. The fixed 20-image test set also explains why the confusion matrices in the present study contain 20 observations rather than all 146 images. Aggregated confusion matrices over all 146 images would require a full outer k-fold validation design, which was not performed in the current analysis.

#### 4.4.5. Exclusion of Imperfect Images

Blurring, tilting, and poor-quality photographs need to be excluded for annotation to be done correctly. Nonetheless, this will limit the practicality of the results since clinical databases are known to have inconsistencies. This study has not analyzed how well the system can perform in situations where it encounters these kinds of images.

#### 4.4.6. Dataset Size

This data set comprises 146 images from one center only, and thus, its applicability and generalization ability is limited. In order to generalize this result and conclude whether such results can be generalized or not, external validation will be required in the form of more data sets from different centers.

### 4.5. Clinical Integration

For this system to be clinically useful, several improvements are necessary. First, features must be normalized to a facial reference frame. Second, the classification task should ideally be reformulated as a regression on deviation magnitude (in millimetres or normalized to interpupillary distance), with subsequent application of a clinically meaningful threshold. This would allow proper statistical evaluation using RMSE, MAE, and Bland–Altman analysis. Third, such a system should serve as a decision-support tool to flag potential deviations for clinician review, rather than as a standalone diagnostic device. Integration with existing digital orthodontic platforms is a potential avenue for future deployment, contingent upon prospective clinical validation [35,36].

Although currently there are several limitations to the system in question, automation is still bringing tangible benefits in terms of time efficiency. By gradually integrating the AI approach into the process, one could decrease the amount of time spent on an orthodontic assessment from 30 min to about 30 s [27,28]. With future developments of such systems becoming Clinical Decision Support Systems, they can increase diagnostics accuracy beyond 93%, thus reducing inconsistencies among physicians [37,38].

### 4.6. Recommendations for Future Work

Based on the limitations identified, the following are recommended for future research: incorporation of a facial reference frame for feature normalization; reformulation of the task as a continuous regression on deviation magnitude; use of a dedicated detection hold-out set or nested cross-validation to prevent information leakage; intra- and inter-observer reliability assessment for ground-truth labels; validation on larger, multi-center datasets including images of varying quality; and exploration of more advanced classifiers such as gradient boosting methods (XGBoost, LightGBM) and lightweight neural network classifier.

The highest methodological priority for future work is leakage-free validation of the complete pipeline. This should involve either re-training YOLOv8 only on the training subset before fixed test-set evaluation or implementing outer k-fold/nested cross-validation so that all 146 images, or all images in a larger future dataset, serve once as detector-unseen and classifier-unseen test cases.

## 5. Conclusions

This study demonstrated the technical feasibility of using YOLOv8-derived bounding-box and keypoint features for exploratory assessment of maxillary dental midline deviation on standardized orthodontic frontal photographs. YOLOv8 achieved high internal localization performance under the present experimental conditions; however, this result was obtained from an internal validation split and should not be interpreted as an independent estimate of detector generalization.

The downstream classification of symmetric versus asymmetric midline conditions achieved a highest point estimate of 75% accuracy with Naive Bayes on the fixed 20-image test set. However, because YOLOv8 was trained on all 146 images before feature extraction, the classification metrics cannot be considered unbiased estimates of full-pipeline generalization. In addition, the confidence intervals of the evaluated classifiers overlapped substantially, preventing statistically conclusive ranking of model performance.

The findings should therefore be interpreted as exploratory proof-of-concept evidence for a two-stage hybrid workflow rather than as validation of a clinically deployable model. Future work must address information leakage through detector re-training within each outer fold, incorporate facial reference frame normalization, provide intra- and inter-observer reliability data, and validate the approach on larger, multi-center datasets.

## Figures and Tables

**Table 1:** Distribution of Images used in the Training and Validation Sets.

	Training Set	Test Set
With Dental Midline Deviation	60	12
Without Dental Midline Deviation	66	8

**Table 2:** Comparative Performance of Machine Learning Models.

Model	Precision (Sym)	Recall (Sym)	F1 (Sym)	Precision (Asym)	Recall (Asym)	F1 (Asym)	Macro F1	Weighted F1	Accuracy	95% Wilson CI
Naive Bayes	0.64	0.88	0.74	0.89	0.67	0.76	0.75	0.75	0.75	0.53–0.89
Logistic Regression	0.46	0.75	0.57	0.71	0.42	0.53	0.55	0.54	0.55	0.34–0.74
Random Forest	0.29	0.25	0.27	0.54	0.58	0.56	0.41	0.44	0.45	0.26–0.66
AdaBoost	0.54	0.88	0.67	0.86	0.50	0.63	0.65	0.65	0.65	0.43–0.82
Support Vector Machine	0.40	1.00	0.57	0.00	0.00	0.00	0.29	0.23	0.40	0.22–0.61
K-Nearest Neighbors	0.40	0.50	0.44	0.60	0.50	0.55	0.49	0.51	0.50	0.30–0.70
Extra Trees	0.50	0.50	0.50	0.67	0.67	0.67	0.58	0.60	0.60	0.39–0.78
Logistic Regression (ElasticNet)	0.40	1.00	0.57	0.00	0.00	0.00	0.29	0.23	0.40	0.22–0.61
Logistic Regression (L1)	0.40	1.00	0.57	0.00	0.00	0.00	0.29	0.23	0.40	0.22–0.61
Linear Discriminant Analysis	0.36	0.50	0.42	0.56	0.42	0.48	0.45	0.45	0.45	0.26–0.66
Decision Tree	0.33	0.38	0.35	0.55	0.50	0.52	0.44	0.45	0.45	0.26–0.66

## Data Availability

The data presented in this study are available on request from the corresponding author. The data are not publicly available due to patient privacy considerations.
